# 2-{1-[2,8-Bis(trifluoro­meth­yl)quinolin-4-yl]-3,5,6,7,8,8a-hexa­hydro-1*H*-1,3-oxazolo[3,4-*a*]pyridin-3-yl}phenol

**DOI:** 10.1107/S1600536811022379

**Published:** 2011-06-18

**Authors:** Raoni S. B. Gonçalves, Carlos R. Kaiser, Marcus V. N. de Souza, James L. Wardell, Solange M. S. V. Wardell, Edward R. T. Tiekink

**Affiliations:** a Fundação Oswaldo Cruz, Instituto de Tecnologia, em Fármacos–Farmanguinhos, R. Sizenando Nabuco, 100, Manguinhos, 21041-250, Rio de Janeiro, RJ, Brazil; b Departamento de Química Orgânica, Instituto de Química, Universidade Federal do Rio de Janeiro, CP 68563, 21945-970 Rio de Janeiro, Brazil; c Centro de Desenvolvimento Tecnológico em Saúde (CDTS), Fundação Oswaldo Cruz (FIOCRUZ), Casa Amarela, Campus de Manguinhos, Av. Brasil 4365, 21040-900 Rio de Janeiro, RJ, Brazil; d CHEMSOL, 1 Harcourt Road, Aberdeen AB15 5NY, Scotland; e Department of Chemistry, University of Malaya, 50603 Kuala Lumpur, Malaysia

## Abstract

In the title mefloquine–oxazolidine derivative, C_24_H_20_F_6_N_2_O_2_, the oxazoline ring adopts an envelope conformation (the flap atom is N) and the piperidine ring has a chair conformation. The oxazoline and benzene residues lie away from the C_6_ ring of the quinoline group and, to a first approximation, to one side of the plane through the ten atoms (r.m.s. deviation = 0.025 Å). An intra­molecular O—H⋯N(piperidine) hydrogen bond is present. The crystal packing features C—H⋯O, C—H⋯F and C—H⋯π(hy­droxy­benzene) inter­actions.

## Related literature

For background to the anti-mycobacterial activities of quinoline derivatives related to mefloquine, see: Gonçalves *et al.* (2010 ▶). For additional geometric analysis, see: Cremer & Pople (1975 ▶); Spek (2009 ▶).
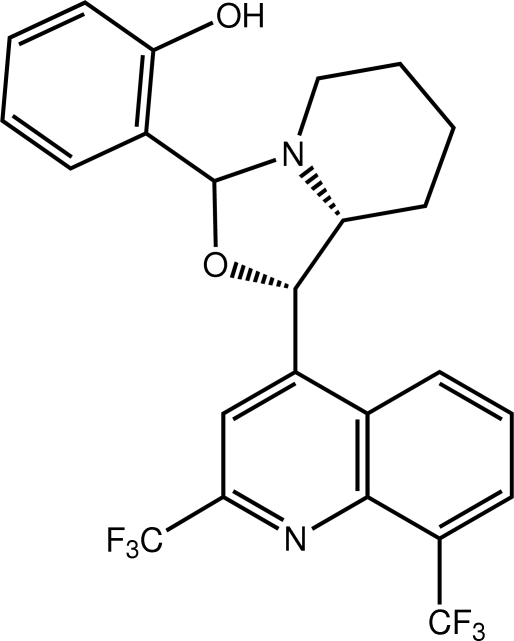


## Experimental

### 

#### Crystal data

C_24_H_20_F_6_N_2_O_2_
*M* *_r_* = 482.42
Orthorhombic, 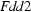

*a* = 27.2766 (11) Å
*b* = 34.1005 (9) Å
*c* = 9.4431 (2) Å
*V* = 8783.5 (5) Å^3^
*Z* = 16
Mo *K*α radiation
μ = 0.13 mm^−1^
*T* = 120 K
0.40 × 0.20 × 0.16 mm

#### Data collection

Enraf–Nonius KappaCCD diffractometer
Absorption correction: multi-scan (*SADABS*; Sheldrick, 2007 ▶) *T*_min_ = 0.799, *T*_max_ = 1.000
13970 measured reflections
2660 independent reflections
2519 reflections with *I* > 2σ(*I*)
*R*_int_ = 0.043

#### Refinement

*R*[*F* ^2^ > 2σ(*F* ^2^)] = 0.035
*wR*(*F* ^2^) = 0.082
*S* = 1.10
2660 reflections
308 parameters
1 restraint
H-atom parameters constrained
Δρ_max_ = 0.20 e Å^−3^
Δρ_min_ = −0.24 e Å^−3^

### 

Data collection: *COLLECT* (Hooft, 1998 ▶); cell refinement: *DENZO* (Otwinowski & Minor, 1997 ▶) and *COLLECT*; data reduction: *DENZO* and *COLLECT*; program(s) used to solve structure: *SHELXS97* (Sheldrick, 2008 ▶); program(s) used to refine structure: *SHELXL97* (Sheldrick, 2008 ▶); molecular graphics: *ORTEP-3* (Farrugia, 1997 ▶) and *DIAMOND* (Brandenburg, 2006 ▶); software used to prepare material for publication: *PLATON* (Spek, 2009 ▶) and *publCIF* (Westrip, 2010 ▶).

## Supplementary Material

Crystal structure: contains datablock(s) global, I. DOI: 10.1107/S1600536811022379/hb5905sup1.cif

Structure factors: contains datablock(s) I. DOI: 10.1107/S1600536811022379/hb5905Isup2.hkl

Additional supplementary materials:  crystallographic information; 3D view; checkCIF report

## Figures and Tables

**Table 1:** Hydrogen-bond geometry (Å, °) *Cg*1 is the centroid of the benzene ring C19–C24.

*D*—H⋯*A*	*D*—H	H⋯*A*	*D*⋯*A*	*D*—H⋯*A*
O2—H2*o*⋯N2	0.84	1.93	2.672 (3)	146
C6—H6⋯O1^i^	0.95	2.52	3.384 (3)	152
C16—H16*B*⋯F4^ii^	0.99	2.47	3.043 (3)	116
C18—H18*B*⋯F1^ii^	0.99	2.54	3.275 (3)	131
C15—H15*B*⋯*Cg*1^iii^	0.99	2.93	3.792 (3)	146

Symmetry codes: (i) 
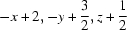
; (ii) 
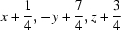
; (iii) 
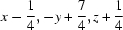
.

**Fig. 1.**
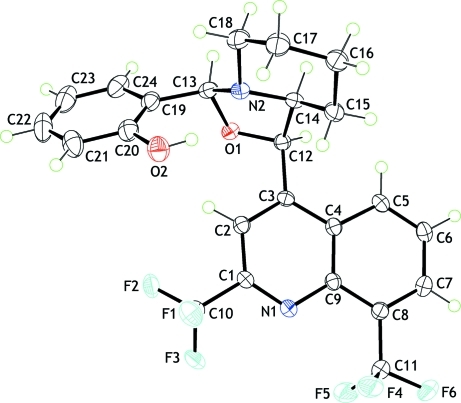
The molecular structure of (I) showing displacement ellipsoids at the 50% probability level.

**Fig. 2.**
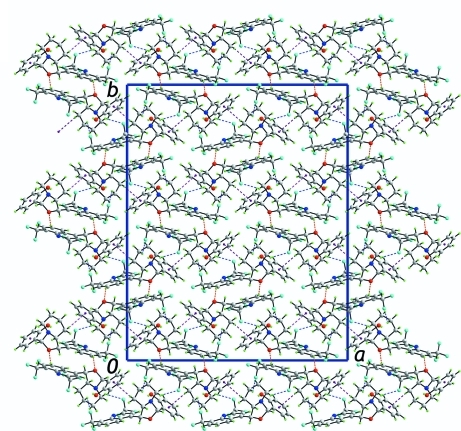
A view in projection down the c axis of the unit-cell contents of (I) with the C—H···O, C—H···F and C—H···π(hydroxybenzene) interactions shown as orange blue and purple dashed lines, respectively.

## supplementary crystallographic information

### Comment

A recent publication reported the synthesis and anti-tubercular activity of mefloquine-oxazolidine derivatives (Gonçalves *et al.*, 2010). Subsequently, crystals became available for one of the derivatives, the title compound (I), allowing full characterization by X-ray crystallography.

In (I), Fig. 1, the oxazoline ring adopts an envelope conformation with the flap atom being N2 as seen in the puckering parameters Q(2) = 0.409 (2) Å and φ_2_ = 100.6 (3) ° (Cremer & Pople, 1975). The piperidinyl ring is close to a chair conformation with puckering parameters: Q(2) = 0.060 (3) Å and Q(3) = -0.596 (3) Å, and amplitudes: Q = 0.599 (3) Å, θ = 174.6 (3) ° and φ = 214 (3) ° (Cremer & Pople, 1975). The 10 non-hydrogen atom comprising the quinoline residue are co-planar with the r.m.s. deviation being 0.025 Å. With reference to this plane, the oxazolidine residue, with the exception of the O1 atom, lies to one side of the plane. By contrast, the benzene ring is somewhat splayed [forming a dihedral angle of 50.34 (11) °] with half the ring above and the other half below the plane through the quinoline atoms. The oxazolidine and benzene ring are directed away from the C_6_ ring of the quinoline residue, and the hydroxyl group is orientated to allow the formation of a O—H···N hydrogen bond, Table 1.

Molecules are stabilized in the crystal structure by a combination of C—H···O, C—H···F and C—H···π(hydroxybenzene) interactions, Table 1 and Fig. 2.

### Experimental

The compound was prepared as reported in the literature (Gonçalves *et al.*, 2010) and was recrystallized from its ethanol solution for the structural study.

### Refinement

The C-bound H atoms were geometrically placed (C—H = 0.95–1.00 Å) and refined as riding with *U*_iso_(H) = 1.2*U*_eq_(C). The O-bound atom was treated similarly with O—H = 0.84 Å, and with *U*_iso_(H) = 1.5*U*_eq_(O). In the absence of significant anomalous scattering effects, 2163 Friedel pairs were averaged in the final refinement. The stereochemistries at the chiral centres were chosen to match the starting mefloquine reagent (Gonçalves *et al.*, 2010).

### Crystal data

**Table:**

C_24_H_20_F_6_N_2_O_2_	*F*(000) = 3968
*M**_r_* = 482.42	*D*_x_ = 1.459 Mg m^−^^3^
Orthorhombic, *F**d**d*2	Mo *K*α radiation, λ = 0.71073 Å
Hall symbol: F 2 -2d	Cell parameters from 11047 reflections
*a* = 27.2766 (11) Å	θ = 2.9–27.5°
*b* = 34.1005 (9) Å	µ = 0.13 mm^−^^1^
*c* = 9.4431 (2) Å	*T* = 120 K
*V* = 8783.5 (5) Å^3^	Block, colourless
*Z* = 16	0.40 × 0.20 × 0.16 mm

### Data collection

**Table:**

Enraf–Nonius KappaCCD diffractometer	2660 independent reflections
Radiation source: Enraf–Nonius FR591 rotating anode	2519 reflections with *I* > 2σ(*I*)
10 cm confocal mirrors	*R*_int_ = 0.043
Detector resolution: 9.091 pixels mm^-1^	θ_max_ = 27.5°, θ_min_ = 3.2°
φ and ω scans	*h* = −35→32
Absorption correction: multi-scan (*SADABS*; Sheldrick, 2007)	*k* = −44→32
*T*_min_ = 0.799, *T*_max_ = 1.000	*l* = −11→12
13970 measured reflections	

### Refinement

**Table:**

Refinement on *F*^2^	Secondary atom site location: difference Fourier map
Least-squares matrix: full	Hydrogen site location: inferred from neighbouring sites
*R*[*F*^2^ > 2σ(*F*^2^)] = 0.035	H-atom parameters constrained
*wR*(*F*^2^) = 0.082	*w* = 1/[σ^2^(*F*_o_^2^) + (0.0295*P*)^2^ + 15.365*P*] where *P* = (*F*_o_^2^ + 2*F*_c_^2^)/3
*S* = 1.10	(Δ/σ)_max_ = 0.001
2660 reflections	Δρ_max_ = 0.20 e Å^−^^3^
308 parameters	Δρ_min_ = −0.24 e Å^−^^3^
1 restraint	Absolute structure: nd
Primary atom site location: structure-invariant direct methods	

### Special details

**Table:**

Geometry. All s.u.'s (except the s.u. in the dihedral angle between two l.s. planes) are estimated using the full covariance matrix. The cell s.u.'s are taken into account individually in the estimation of s.u.'s in distances, angles and torsion angles; correlations between s.u.'s in cell parameters are only used when they are defined by crystal symmetry. An approximate (isotropic) treatment of cell s.u.'s is used for estimating s.u.'s involving l.s. planes.
Refinement. Refinement of *F*^2^ against ALL reflections. The weighted *R*-factor *wR* and goodness of fit *S* are based on *F*^2^, conventional *R*-factors *R* are based on *F*, with *F* set to zero for negative *F*^2^. The threshold expression of *F*^2^ > 2σ(*F*^2^) is used only for calculating *R*-factors(gt) *etc*. and is not relevant to the choice of reflections for refinement. *R*-factors based on *F*^2^ are statistically about twice as large as those based on *F*, and *R*- factors based on ALL data will be even larger.

### Fractional atomic coordinates and isotropic or equivalent isotropic displacement parameters (Å^2^)

**Table:**

	*x*	*y*	*z*	*U*_iso_*/*U*_eq_	
F1	0.98349 (6)	0.85851 (4)	0.25475 (17)	0.0325 (3)	
F2	1.03760 (5)	0.81329 (5)	0.22731 (16)	0.0352 (4)	
F3	0.96194 (6)	0.80141 (5)	0.18235 (15)	0.0332 (4)	
F4	0.83400 (6)	0.81192 (5)	0.47423 (18)	0.0387 (4)	
F5	0.85056 (6)	0.75173 (5)	0.4262 (2)	0.0435 (4)	
F6	0.79918 (6)	0.76574 (6)	0.59082 (19)	0.0433 (4)	
O1	1.10450 (6)	0.78733 (5)	0.67372 (18)	0.0214 (3)	
O2	1.10905 (7)	0.87646 (6)	0.4756 (2)	0.0331 (4)	
H2O	1.0968	0.8720	0.5556	0.050*	
N1	0.93890 (7)	0.79834 (6)	0.4583 (2)	0.0203 (4)	
N2	1.10727 (7)	0.85110 (6)	0.7433 (2)	0.0226 (4)	
C1	0.98410 (8)	0.80770 (6)	0.4242 (2)	0.0187 (4)	
C2	1.02460 (8)	0.80730 (6)	0.5156 (2)	0.0198 (4)	
H2	1.0563	0.8139	0.4816	0.024*	
C3	1.01759 (8)	0.79725 (6)	0.6548 (2)	0.0176 (4)	
C4	0.96894 (8)	0.78816 (6)	0.7003 (2)	0.0189 (4)	
C5	0.95739 (9)	0.77823 (7)	0.8426 (3)	0.0214 (5)	
H5	0.9824	0.7786	0.9125	0.026*	
C6	0.91067 (9)	0.76812 (7)	0.8798 (3)	0.0233 (5)	
H6	0.9034	0.7616	0.9753	0.028*	
C7	0.87321 (9)	0.76741 (7)	0.7767 (3)	0.0242 (5)	
H7	0.8409	0.7600	0.8033	0.029*	
C8	0.88277 (8)	0.77726 (7)	0.6394 (3)	0.0212 (5)	
C9	0.93106 (8)	0.78838 (6)	0.5973 (2)	0.0186 (4)	
C10	0.99188 (8)	0.82002 (7)	0.2718 (3)	0.0236 (5)	
C11	0.84193 (9)	0.77694 (8)	0.5328 (3)	0.0273 (5)	
C12	1.06091 (8)	0.79426 (7)	0.7548 (2)	0.0201 (4)	
H12	1.0556	0.7719	0.8218	0.024*	
C13	1.13849 (8)	0.81902 (7)	0.6950 (3)	0.0231 (5)	
H13	1.1626	0.8119	0.7707	0.028*	
C14	1.07232 (8)	0.83156 (7)	0.8391 (3)	0.0224 (5)	
H14	1.0902	0.8239	0.9273	0.027*	
C15	1.03185 (9)	0.85983 (7)	0.8790 (3)	0.0257 (5)	
H15A	1.0080	0.8467	0.9422	0.031*	
H15B	1.0143	0.8687	0.7930	0.031*	
C16	1.05498 (10)	0.89492 (8)	0.9549 (3)	0.0313 (6)	
H16A	1.0294	0.9147	0.9750	0.038*	
H16B	1.0690	0.8862	1.0463	0.038*	
C17	1.09526 (10)	0.91343 (8)	0.8645 (3)	0.0341 (6)	
H17A	1.0803	0.9258	0.7801	0.041*	
H17B	1.1119	0.9342	0.9198	0.041*	
C18	1.13303 (9)	0.88317 (8)	0.8172 (3)	0.0310 (6)	
H18A	1.1572	0.8955	0.7528	0.037*	
H18B	1.1508	0.8727	0.9004	0.037*	
C19	1.16494 (9)	0.82626 (7)	0.5575 (3)	0.0262 (5)	
C20	1.14874 (9)	0.85282 (7)	0.4556 (3)	0.0287 (5)	
C21	1.17304 (11)	0.85539 (8)	0.3246 (3)	0.0372 (7)	
H21	1.1618	0.8734	0.2550	0.045*	
C22	1.21281 (12)	0.83207 (9)	0.2970 (4)	0.0431 (7)	
H22	1.2291	0.8341	0.2083	0.052*	
C23	1.22951 (11)	0.80566 (9)	0.3970 (4)	0.0417 (7)	
H23	1.2570	0.7895	0.3770	0.050*	
C24	1.20596 (10)	0.80299 (8)	0.5263 (3)	0.0331 (6)	
H24	1.2178	0.7851	0.5953	0.040*	

### Atomic displacement parameters (Å^2^)

**Table:**

	*U*^11^	*U*^22^	*U*^33^	*U*^12^	*U*^13^	*U*^23^
F1	0.0439 (9)	0.0275 (8)	0.0262 (8)	0.0023 (6)	−0.0006 (7)	0.0088 (6)
F2	0.0274 (7)	0.0554 (10)	0.0227 (8)	0.0085 (7)	0.0077 (6)	0.0121 (7)
F3	0.0377 (9)	0.0460 (9)	0.0160 (7)	−0.0085 (7)	−0.0028 (6)	−0.0014 (7)
F4	0.0344 (8)	0.0471 (9)	0.0347 (9)	0.0040 (7)	−0.0083 (7)	0.0118 (8)
F5	0.0339 (8)	0.0578 (11)	0.0387 (9)	−0.0042 (8)	−0.0057 (8)	−0.0217 (9)
F6	0.0202 (7)	0.0723 (12)	0.0373 (10)	−0.0094 (7)	0.0010 (7)	0.0083 (9)
O1	0.0193 (8)	0.0214 (7)	0.0236 (8)	−0.0016 (6)	−0.0005 (7)	−0.0004 (7)
O2	0.0363 (10)	0.0313 (9)	0.0316 (10)	0.0013 (8)	0.0036 (8)	0.0049 (8)
N1	0.0217 (9)	0.0222 (9)	0.0170 (10)	−0.0002 (7)	0.0003 (8)	−0.0004 (7)
N2	0.0221 (9)	0.0236 (9)	0.0222 (10)	−0.0038 (7)	−0.0015 (8)	−0.0026 (8)
C1	0.0217 (11)	0.0182 (10)	0.0162 (10)	0.0003 (8)	0.0000 (9)	0.0001 (8)
C2	0.0211 (10)	0.0186 (10)	0.0196 (11)	−0.0006 (8)	0.0007 (9)	0.0009 (9)
C3	0.0197 (10)	0.0155 (9)	0.0175 (11)	−0.0002 (8)	−0.0019 (8)	−0.0010 (8)
C4	0.0218 (10)	0.0166 (9)	0.0184 (11)	0.0000 (8)	0.0015 (9)	0.0014 (9)
C5	0.0266 (12)	0.0213 (11)	0.0164 (11)	0.0000 (9)	0.0002 (9)	0.0009 (9)
C6	0.0270 (12)	0.0240 (11)	0.0188 (11)	0.0036 (9)	0.0044 (9)	0.0026 (9)
C7	0.0213 (11)	0.0238 (11)	0.0275 (12)	0.0017 (9)	0.0054 (10)	0.0017 (10)
C8	0.0205 (11)	0.0217 (11)	0.0215 (11)	−0.0003 (9)	0.0005 (9)	−0.0002 (9)
C9	0.0205 (10)	0.0173 (10)	0.0180 (11)	−0.0002 (8)	0.0013 (9)	−0.0008 (8)
C10	0.0233 (11)	0.0278 (12)	0.0197 (11)	−0.0008 (9)	−0.0010 (10)	0.0005 (10)
C11	0.0210 (11)	0.0351 (13)	0.0257 (12)	−0.0012 (10)	0.0014 (10)	−0.0002 (10)
C12	0.0205 (10)	0.0209 (10)	0.0189 (11)	−0.0018 (8)	−0.0006 (9)	0.0014 (9)
C13	0.0192 (10)	0.0248 (11)	0.0252 (13)	−0.0023 (8)	−0.0043 (9)	0.0010 (9)
C14	0.0223 (11)	0.0273 (11)	0.0175 (11)	−0.0033 (9)	−0.0035 (9)	−0.0019 (9)
C15	0.0280 (12)	0.0258 (12)	0.0232 (12)	−0.0033 (9)	0.0013 (10)	−0.0053 (10)
C16	0.0319 (13)	0.0308 (13)	0.0311 (14)	−0.0035 (10)	0.0018 (11)	−0.0109 (11)
C17	0.0362 (14)	0.0282 (13)	0.0379 (15)	−0.0092 (10)	0.0037 (12)	−0.0117 (11)
C18	0.0281 (12)	0.0311 (13)	0.0338 (14)	−0.0078 (10)	−0.0015 (11)	−0.0068 (11)
C19	0.0237 (12)	0.0261 (12)	0.0287 (13)	−0.0051 (9)	0.0027 (10)	−0.0030 (10)
C20	0.0310 (13)	0.0243 (11)	0.0307 (14)	−0.0067 (9)	0.0035 (11)	−0.0017 (10)
C21	0.0514 (17)	0.0286 (13)	0.0316 (15)	−0.0107 (12)	0.0096 (13)	0.0003 (12)
C22	0.0508 (17)	0.0393 (16)	0.0391 (17)	−0.0136 (13)	0.0220 (14)	−0.0087 (13)
C23	0.0344 (15)	0.0373 (15)	0.0535 (19)	−0.0066 (13)	0.0163 (14)	−0.0119 (14)
C24	0.0265 (12)	0.0285 (13)	0.0444 (16)	−0.0030 (10)	0.0034 (12)	−0.0038 (12)

### Geometric parameters (Å, °)

**Table:**

F1—C10	1.342 (3)	C8—C9	1.427 (3)
F2—C10	1.336 (3)	C8—C11	1.502 (3)
F3—C10	1.336 (3)	C12—C14	1.532 (3)
F4—C11	1.332 (3)	C12—H12	1.0000
F5—C11	1.344 (3)	C13—C19	1.506 (3)
F6—C11	1.344 (3)	C13—H13	1.0000
O1—C12	1.434 (3)	C14—C15	1.514 (3)
O1—C13	1.438 (3)	C14—H14	1.0000
O2—C20	1.363 (3)	C15—C16	1.530 (3)
O2—H2O	0.8400	C15—H15A	0.9900
N1—C1	1.314 (3)	C15—H15B	0.9900
N1—C9	1.372 (3)	C16—C17	1.528 (4)
N2—C13	1.460 (3)	C16—H16A	0.9900
N2—C14	1.473 (3)	C16—H16B	0.9900
N2—C18	1.475 (3)	C17—C18	1.525 (4)
C1—C2	1.401 (3)	C17—H17A	0.9900
C1—C10	1.514 (3)	C17—H17B	0.9900
C2—C3	1.372 (3)	C18—H18A	0.9900
C2—H2	0.9500	C18—H18B	0.9900
C3—C4	1.429 (3)	C19—C20	1.393 (4)
C3—C12	1.516 (3)	C19—C24	1.403 (4)
C4—C9	1.419 (3)	C20—C21	1.406 (4)
C4—C5	1.421 (3)	C21—C22	1.370 (4)
C5—C6	1.366 (3)	C21—H21	0.9500
C5—H5	0.9500	C22—C23	1.382 (5)
C6—C7	1.411 (3)	C22—H22	0.9500
C6—H6	0.9500	C23—C24	1.382 (4)
C7—C8	1.364 (3)	C23—H23	0.9500
C7—H7	0.9500	C24—H24	0.9500
			
C12—O1—C13	109.65 (17)	N2—C13—C19	115.2 (2)
C20—O2—H2O	109.5	O1—C13—H13	110.0
C1—N1—C9	116.2 (2)	N2—C13—H13	110.0
C13—N2—C14	103.32 (18)	C19—C13—H13	110.0
C13—N2—C18	115.18 (19)	N2—C14—C15	109.69 (19)
C14—N2—C18	110.72 (19)	N2—C14—C12	100.86 (18)
N1—C1—C2	125.9 (2)	C15—C14—C12	120.68 (19)
N1—C1—C10	115.6 (2)	N2—C14—H14	108.3
C2—C1—C10	118.5 (2)	C15—C14—H14	108.3
C3—C2—C1	118.8 (2)	C12—C14—H14	108.3
C3—C2—H2	120.6	C14—C15—C16	108.3 (2)
C1—C2—H2	120.6	C14—C15—H15A	110.0
C2—C3—C4	118.1 (2)	C16—C15—H15A	110.0
C2—C3—C12	120.3 (2)	C14—C15—H15B	110.0
C4—C3—C12	121.48 (19)	C16—C15—H15B	110.0
C9—C4—C5	119.2 (2)	H15A—C15—H15B	108.4
C9—C4—C3	118.0 (2)	C17—C16—C15	111.0 (2)
C5—C4—C3	122.8 (2)	C17—C16—H16A	109.4
C6—C5—C4	120.7 (2)	C15—C16—H16A	109.4
C6—C5—H5	119.7	C17—C16—H16B	109.4
C4—C5—H5	119.7	C15—C16—H16B	109.4
C5—C6—C7	120.1 (2)	H16A—C16—H16B	108.0
C5—C6—H6	119.9	C18—C17—C16	111.7 (2)
C7—C6—H6	119.9	C18—C17—H17A	109.3
C8—C7—C6	120.8 (2)	C16—C17—H17A	109.3
C8—C7—H7	119.6	C18—C17—H17B	109.3
C6—C7—H7	119.6	C16—C17—H17B	109.3
C7—C8—C9	120.4 (2)	H17A—C17—H17B	107.9
C7—C8—C11	119.6 (2)	N2—C18—C17	108.6 (2)
C9—C8—C11	120.0 (2)	N2—C18—H18A	110.0
N1—C9—C4	122.9 (2)	C17—C18—H18A	110.0
N1—C9—C8	118.4 (2)	N2—C18—H18B	110.0
C4—C9—C8	118.7 (2)	C17—C18—H18B	110.0
F3—C10—F2	106.9 (2)	H18A—C18—H18B	108.4
F3—C10—F1	106.50 (19)	C20—C19—C24	118.4 (2)
F2—C10—F1	106.81 (19)	C20—C19—C13	123.4 (2)
F3—C10—C1	112.58 (19)	C24—C19—C13	118.1 (2)
F2—C10—C1	112.49 (19)	O2—C20—C19	122.7 (2)
F1—C10—C1	111.2 (2)	O2—C20—C21	117.4 (2)
F4—C11—F6	106.4 (2)	C19—C20—C21	119.9 (2)
F4—C11—F5	106.9 (2)	C22—C21—C20	120.3 (3)
F6—C11—F5	106.0 (2)	C22—C21—H21	119.9
F4—C11—C8	113.1 (2)	C20—C21—H21	119.9
F6—C11—C8	111.8 (2)	C21—C22—C23	120.6 (3)
F5—C11—C8	112.1 (2)	C21—C22—H22	119.7
O1—C12—C3	108.97 (18)	C23—C22—H22	119.7
O1—C12—C14	104.21 (17)	C22—C23—C24	119.5 (3)
C3—C12—C14	115.21 (19)	C22—C23—H23	120.2
O1—C12—H12	109.4	C24—C23—H23	120.2
C3—C12—H12	109.4	C23—C24—C19	121.2 (3)
C14—C12—H12	109.4	C23—C24—H24	119.4
O1—C13—N2	103.34 (18)	C19—C24—H24	119.4
O1—C13—C19	108.15 (19)		
			
C9—N1—C1—C2	1.9 (3)	C2—C3—C12—O1	−23.2 (3)
C9—N1—C1—C10	−177.92 (19)	C4—C3—C12—O1	154.04 (19)
N1—C1—C2—C3	−1.7 (3)	C2—C3—C12—C14	93.5 (3)
C10—C1—C2—C3	178.2 (2)	C4—C3—C12—C14	−89.3 (3)
C1—C2—C3—C4	−0.8 (3)	C12—O1—C13—N2	−21.6 (2)
C1—C2—C3—C12	176.54 (19)	C12—O1—C13—C19	−144.21 (19)
C2—C3—C4—C9	2.7 (3)	C14—N2—C13—O1	40.1 (2)
C12—C3—C4—C9	−174.6 (2)	C18—N2—C13—O1	160.9 (2)
C2—C3—C4—C5	−178.6 (2)	C14—N2—C13—C19	157.82 (19)
C12—C3—C4—C5	4.1 (3)	C18—N2—C13—C19	−81.3 (3)
C9—C4—C5—C6	1.5 (3)	C13—N2—C14—C15	−170.42 (19)
C3—C4—C5—C6	−177.2 (2)	C18—N2—C14—C15	65.7 (2)
C4—C5—C6—C7	0.0 (4)	C13—N2—C14—C12	−42.1 (2)
C5—C6—C7—C8	−0.9 (4)	C18—N2—C14—C12	−165.92 (19)
C6—C7—C8—C9	0.2 (4)	O1—C12—C14—N2	28.6 (2)
C6—C7—C8—C11	−178.9 (2)	C3—C12—C14—N2	−90.8 (2)
C1—N1—C9—C4	0.3 (3)	O1—C12—C14—C15	149.4 (2)
C1—N1—C9—C8	−178.9 (2)	C3—C12—C14—C15	30.1 (3)
C5—C4—C9—N1	178.7 (2)	N2—C14—C15—C16	−60.6 (3)
C3—C4—C9—N1	−2.6 (3)	C12—C14—C15—C16	−177.0 (2)
C5—C4—C9—C8	−2.1 (3)	C14—C15—C16—C17	54.6 (3)
C3—C4—C9—C8	176.6 (2)	C15—C16—C17—C18	−53.1 (3)
C7—C8—C9—N1	−179.5 (2)	C13—N2—C18—C17	−178.2 (2)
C11—C8—C9—N1	−0.4 (3)	C14—N2—C18—C17	−61.5 (3)
C7—C8—C9—C4	1.3 (3)	C16—C17—C18—N2	55.2 (3)
C11—C8—C9—C4	−179.6 (2)	O1—C13—C19—C20	91.1 (3)
N1—C1—C10—F3	−32.8 (3)	N2—C13—C19—C20	−23.9 (3)
C2—C1—C10—F3	147.4 (2)	O1—C13—C19—C24	−84.1 (3)
N1—C1—C10—F2	−153.6 (2)	N2—C13—C19—C24	160.9 (2)
C2—C1—C10—F2	26.6 (3)	C24—C19—C20—O2	179.5 (2)
N1—C1—C10—F1	86.7 (2)	C13—C19—C20—O2	4.3 (4)
C2—C1—C10—F1	−93.2 (2)	C24—C19—C20—C21	0.8 (4)
C7—C8—C11—F4	119.2 (3)	C13—C19—C20—C21	−174.4 (2)
C9—C8—C11—F4	−59.9 (3)	O2—C20—C21—C22	−179.1 (3)
C7—C8—C11—F6	−0.9 (3)	C19—C20—C21—C22	−0.4 (4)
C9—C8—C11—F6	179.9 (2)	C20—C21—C22—C23	0.2 (5)
C7—C8—C11—F5	−119.8 (3)	C21—C22—C23—C24	−0.4 (5)
C9—C8—C11—F5	61.0 (3)	C22—C23—C24—C19	0.8 (4)
C13—O1—C12—C3	118.85 (19)	C20—C19—C24—C23	−1.0 (4)
C13—O1—C12—C14	−4.6 (2)	C13—C19—C24—C23	174.4 (2)

### Hydrogen-bond geometry (Å, °)

**Table:**

Cg1 is the centroid of the benzene ring C19–C24.

**Table:**

*D*—H···*A*	*D*—H	H···*A*	*D*···*A*	*D*—H···*A*
O2—H2O···N2	0.84	1.93	2.672 (3)	146
C6—H6···O1^i^	0.95	2.52	3.384 (3)	152
C16—H16B···F4^ii^	0.99	2.47	3.043 (3)	116
C18—H18B···F1^ii^	0.99	2.54	3.275 (3)	131
C15—H15B···Cg1^iii^	0.99	2.93	3.792 (3)	146

Symmetry codes: (i) −*x*+2, −*y*+3/2, *z*+1/2; (ii) *x*+1/4, −*y*+7/4, *z*+3/4; (iii) *x*−1/4, −*y*+7/4, *z*+1/4.
